# Allylpyrocatechol, isolated from betel leaf ameliorates thyrotoxicosis in rats by altering thyroid peroxidase and thyrotropin receptors

**DOI:** 10.1038/s41598-019-48653-9

**Published:** 2019-08-22

**Authors:** Sunanda Panda, Malabika Sikdar, Sagarika Biswas, Rajesh Sharma, Anand Kar

**Affiliations:** 1 School of Pharmacy, Devi Ahilya University, Indore, India; 2 Department of Zoology, Dr. Hari Singh Gour Vishwavidyalaya, Saugar, India; 3 Department of Genomics and Molecular Medicine, CSIR-Institute of Genomics and Integrative Biology, New Delhi, India; 4 Thyroid Research laboratory, School of Life Sciences, Devi Ahilya University, Indore, India

**Keywords:** Endocrinology, Thyroid diseases

## Abstract

Allylpyrocatechol (APC) was isolated from betel leaf and its possible role in L-thyroxin (L-T_4_)-induced thyrotoxic rats was evaluated. The disease condition, thyrotoxicosis was confirmed by higher levels of thyroid hormones and low thyrotropin (TSH) in serum. Increased hepatic activities of 5′-mono-deiodinase(5′D1), glucose-6-phospatase (G-6-Pase); serum concentrations of alanine transaminase (ALT), aspartate aminotransferase (AST), lactate dehydrogenase(LDH) and tumour necrosis factor-alpha(TNF-α) were observed in thyrotoxic rats. Hepatic lipid peroxidation(LPO) was also increased and the endogenous antioxidants were depleted in these rats. In western blot analysis thyroid peroxidase expression was found to be reduced, whereas thyrotropin receptor(TSHR) expression was enhanced in thyroid gland of these animals. On the other hand, APC treatment in thyrotoxic rats decreased the levels of serum thyroid hormones, ALT, AST, TNF-α and LDH, as well as hepatic 5′ D1 and G-6-Pase activities. However, it increased the serum TSH levels. APC also reduced the hepatic LPO and increased the cellular antioxidants in thyrotoxic rats. However, expression of TSHR was inhibited and TPO was increased by APC. The test compound also improved histological features in both liver and thyroid. Present report appears to be the first one that indicates the positive role of APC in ameliorating T_4_-induced thyrotoxicosis.

## Introduction

Thyroid hormones, triiodothyronine (T_3_) and thyroxin (T_4_) are known to regulate all most all body functions including development, differentiation and metabolism^1,2^. Compared to T_4_, T_3_ is considered to be more biologically active. While T_4_ is synthesized only in the thyroid gland, about 90% of the total circulating T_3_ is produced by the conversion of T_4_ to T_3_ in the extra-thyroidal tissues, that is catalysed by the enzyme type 1 iodothyronine 5′-deiodinase (5′D-1) in the liver, kidney, and skeletal muscle and to some extent in thyroid gland^3,4^. To assess the thyroid function normally the serum levels of T_3_, T_4_ and TSH are estimated. Low or high levels thyroid hormones in circulation are associated with thyroid dysfunction as well as liver disorder.

Thyrotoxicosis, a condition of thyroid dysfunction is a clinical syndrome of hyper metabolism resulting from increased levels of serum T_4_ and/or T_3_ that affect all most all the physiological systems^5,6^. Thyrotoxicosis can result from a destructive process in the thyroid leading to an unregulated release of stored thyroid hormones without increased production^7^. This problem, if not treated properly may end up in serious abnormalities.

Earlier some investigations were made in thyrotoxic rat model showing marked increase in serum T_3_ and T_4_ levels with a loss of body weight^7–9^. In a recent investigation we have also shown that the experimental thyrotoxic rat model can be developed by the intraperitoneal injection of L-T_4_ (500 μg/kg) once daily for 12 consecutive days and this model was found to be quite suitable to study the effects of bioactive compound^10^.

In recent years, a tremendous increase in the interest has been generated on the identification of novel compounds that may have potential in clinical medicine. However, nothing much had been investigated with respect to the regulation of thyrotoxicosis by plant derived active compounds.

*Piper betel* (family, Piperaceae) is a widely cultivated plant in the tropical and subtropical regions of the world, particularly in India, Srilanka, Malaysia, Thailand, Taiwan and other South-east Asian countries. Although its leaves are used as betel quid, it also possesses hepatoprotective, antiulcer, antioxidative and anti-inflammatory activities^11,12^. However, till date nothing has been reported on the thyroid regulating potential of any of its bioactive compounds. Allylpyrocatechol (APC), a major phenolic constituent of betel leaf was reported to posses hepatoprotective, anti-inflammatory, antioxidative, and antiulcer activities^13–17^. As in our earlier study, betel leaf crude extract was found to be thyroid-inhibitory in nature^18^; it was presumed that there could be an active compound in betel leaf that can regulate thyroid dysfunction. In this investigation we have isolated allylpyrocatechol the main phenolic compound of betel leaf and evaluated its thyroid inhibitory effects, if any, in T_4_-induced thyrotoxic rats. We also compared its activity with that of a conventional thyroid inhibitory drug, propylthiouracil (PTU) that primarily inhibits synthesis of thyroid hormones by reducing organification of iodide and coupling of iodothyronines^19^. PTU also decreases the conversion of T_4_ to T_3_ in peripheral tissues by inhibiting the outer ring deiodination of T_4_^20^. Towards better understanding on the mode of action of the test compound, expressions of thyroid peroxidase and thyrotropin receptor were also examined.

## Results

### Isolated compound and its characterization

**Figure 1:**
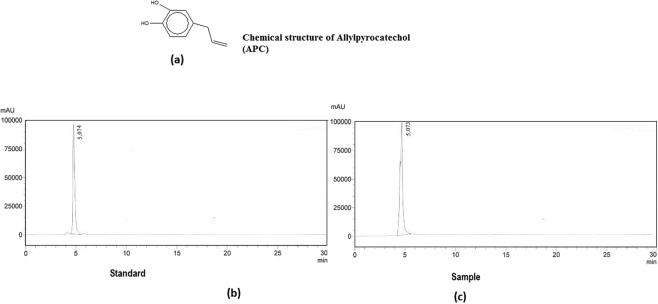
(**a**) Chemical structure of allylpyrocatechol (APC), isolated from *Piper betel* leaf extract. (**b**) HPLC chromatogram of standard APC with retention time of 5.074 minutes. **(c**) HPLC chromatogram of sample APC showing retention time at 5.073 min. It nearly matches with that of the standard.

On the basis of spectroscopic analyses, the compound (Fig. 1a) was identified as allylpyrocatechol. This compound was obtained as light yellow oil with a yield of 0.92% (w/w) and its molecular weight was deduced from gas chromatography mass spectroscopy (GC-MS) at m/z as 150.17. ^1^H NMR and ^13^C NMR data are in full agreement with the proposed structure of APC^21^. Purity of APC was checked by high-performance liquid chromatography (HPLC) and was found to be 98.2%. Figure 1b,c show the HPLC chromatogram.

**Figure 2:**
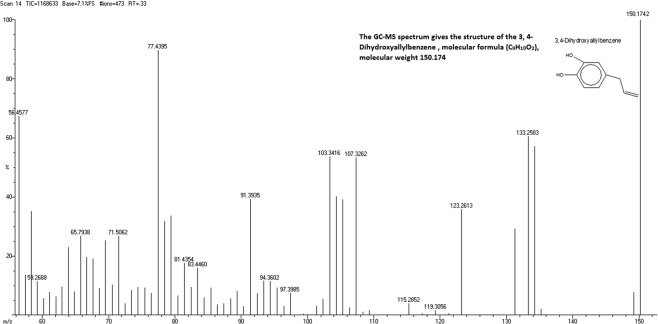
The GC-MS spectrum showing the structure of the APC, molecular formula (C_9_H_10_O_2_) and molecular mass, 150.174.

Prediction of the chemical structure and formula of APC was made from the GC-MS spectrum (Fig. 2). Based on the fragmentation route, the molecular formula was deduced as C_9_H_10_O_2_. According to the database (library search report NIST05), the high similarity (99%) was achieved for the compound with chemical name 3,4-Dihydroxyallylbenzene. Some more characteristics of APC are: IR = 3309, 1606.5, 1408.85, 1272.41, 1184.26, 909.63, 863.34, 784.47 cm^−1^; ^1^H NMR: _δ_3.29 (d, *J* = 6.5 Hz), 2 H, ArCH2), 4.99–5.04(m, 2 H, olefin), 5.36 (broad, 2 H, 2xAr-OH) and 5.08–6.01(m, 1 H, olefin, 6.56–6.76) and ^13^CNMR (CDCl^3^, 50 MHz): _δ_39.33, 115.28, 115.77, 122.65, 131.98, 132.84, 140.77, 143.1. The IR and NMR (^13^C and ^1^H) spectra are given in Supplementary Section (Fig. S1a–c).

### Changes in body weight

While in T_4_- treated thyrotoxic animals, a significant decrease in final body weight (b.wt.) was observed (by 23.24%), no significant differences were seen between initial and final b.wt. of animals in control, T_4_ + APC and in T_4_ + PTU groups. However, it was significantly increased (by 8.36%) in animals treated with APC only (Table 1).

**Table 1:** Effects of the test compound, APC on body weight, different serum lipids & liver marker enzymes in the normal and T_4_-induced rats.

Parameters	Cont	APC	T_4_	T_4_ + APC	T_4_ + PTU
**Body weight (g)**					
Initial	171.2 ± 2.17	174.6 ± 2.3	175.4 ± 2.43	172.5 ± 2.61	173.7 ± 2.52
Final	176.7 ± 1.21	189.2 ± 3.41^b^	135.3 ± 1.69^a^	166.4 ± 3.12	165.6 ± 3.67
**Lipid profile**					
TC (mg/dl)	86.1 ± 2.30	92.4 ± 1.92	53.21 ± 1.42^a^	90.6 ± 2.51^x^	92.7 ± 3.76^x^
TG (mg/dl)	94.2 ± 3.98	84.6 ± 4.70	52.7 ± 1.54^a^	92.71 ± 2.26^x^	95.9 ± 3.91^x^
HDL-C (mg/dl)	31.6 ± 0.70	33.5 ± 1.95	21.7 ± 0.76^b^	30.1 ± 1.83^y^	29.8 ± 1.47^z^
VLDL-C (mg/dl)	25.2 ± 1.71	23.6 ± 1.84	12.7 ± 0.81^a^	26.2 ± 1.71^x^	22.9 ± 3.76^y^
LDL-C (mg/dl)	31.7 ± 1.76	34.4 ± 1.27	18.6 ± 1.27^a^	38.1 ± 2.34^x^	36.7 ± 3.99^y^
**Liver markers**					
ALT (IU/L)	37.6 ± 1.71	28.3 ± 1.23^a^	91.5 ± 2.88^a^	49.16 ± 1.67^x^	62.4 ± 3.09^y^
AST (IU/L)	53.4 ± 2.91	38.8 ± 1.75^a^	160.2 ± 3.64^a^	58.7 ± 2.41^x^	119.2 ± 4.98^x^
LDH (IU/L)	86.5 ± 3.12	79.24 ± 3.98	184.7 ± 4.18^a^	96.32 ± 3.57^x^	124.12 ± 5.17^x^
TNFα (pg/ml)	117.26 ± 4.96	109.07 ± 3.98	1418.69 ± 11.87^a^	386.09 ± 5.16^x^	820.40 ± 7.09^x^

Data are in means ± SEM, n = 7. ^a^*P* < 0.001 and ^b^*P* < 0.01 as compared to their initial b.wt. ^a^*P* < 0.001 and ^b^*P* < 0.01, as compared to the respective control value; whereas ^x^*P* < 0.001, ^y^*P* < 0.01 and ^z^*P* < 0.05 as compared to the respective value of thyroxin (T_4_) treated animals.

### Changes in thyroid hormones and 5′deiodinase-1 activity

**Figure 3:**
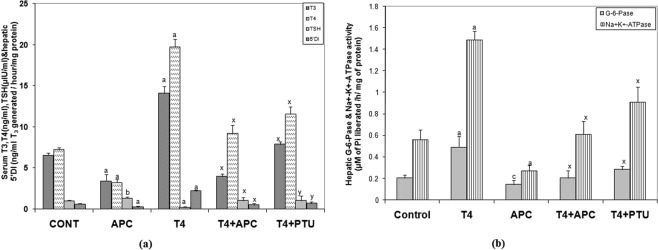
(**a**) Changes in concentrations of serum T_3_ (ng/ml), T_4_ (ng/ml X10), TSH (µIU/ml) and hepatic 5′D1 (ng/ml T_3_ generated/hour/mg protein), following the administration APC (2.0 mg/kg/d) alone or T_4_ + APC or T_4_ + PTU. Each bar represents the mean ± SEM (n = 7). ^a^*P* < 0.001 and ^b^*P* < 0.01 as compared to the respective control value; whereas ^x^*P* < 0.001 and ^y^*P* < 0.01, as compared to the respective value of L-T_4_ treated group. (**b**) Changes in hepatic G-6-Pase (μM of Pi liberated/h/mg protein) and Na^+^-K^+^-ATPase activity (μM of Pi liberated/h/mg of protein) following the administration APC (2.0 mg/kg/d) alone or T_4_ + APC or T_4_ + PTU in rats. Each vertical bar represents the mean ± SEM. (n = 7). ^a^*P* < 0.001 as compared to the respective control value, whereas ^x^*P* < 0.001 as compared to the value of L-T_4_ treated animals.

While in L-T_4_-induced rats there was a significant increase in serum levels of T_3_, T_4_ and in hepatic 5′D1 activity; a decrease in the thyrotropin (TSH) level was noticed, indicating the thyrotoxic condition. However, treatment with APC nearly normalized the serum thyroid hormone concentrations in T_4_-induced rats (Fig. 3a). In PTU + T_4_ treated animals also, thyroid hormones and 5′D1 were inhibited. In euthyroid animals, APC markedly decreased the concentration of both the thyroid hormones and 5′D1 activity and increased the TSH level.

### Changes in hepatic Glucose 6-phosphatase and Sodium Potassium ATPase activities

Following T_4_ administration to euthyroid rats, there was a significant increase in the activities of hepatic glucose 6-phosphatase (G-6-Pase) and sodium potassium ATPase *(*Na^+^-K^+^-ATPase). However, in APC + T_4_ and in PTU + T_4_ treated rats, activities of both the enzymes were inhibited significantly (Fig. 3b). In APC treated animals also, both the enzymes activities were decreased as compared to their respective control value.

### Changes in different serum lipids

As compared to the value of the control group, administration of T_4_ decreased the serum concentrations of total cholesterol (TC), triglyceride (TG), low- density lipoprotein cholesterol (LDL-C), very low-density lipoprotein cholesterol (VLDL-C) and high density lipoprotein cholesterol (HDL-C). However, APC or PTU administration in thyrotoxic rats elevated the levels of these serum lipids near to the normal/control values (Table 1). Only in APC treatment in euthyroid animals, no significant differences were found when compared to the control group.

### Changes in liver enzymes

A marked increase in the levels of serum alanine transaminase (ALT), aspartate amino transferase (AST) and lactate dehydrogenase (LDH) was observed in L-T_4_ treated thyrotoxic rats, whereas, their concentrations were decreased significantly in T_4_ + APC and in T_4_ + PTU treated animals as compared to that of L-T_4_-induced rats (Table 1).

### Changes in inflammatory cytokine in the serum

Compared to the value of the control animals, serum level of tumor necrosis factor-alpha (TNF-α**)** was markedly elevated in thyrotoxic rats. But the treatment of T_4_-induced animals with APC reduced it significantly. In PTU + T_4_ treated group also, level of TNF-α decreased significantly. In APC alone treated group, the value was nearly the same to that of control group (Table 1).

### Changes in hepatic lipid peroxidation and in antioxidant levels

**Figure 4:**
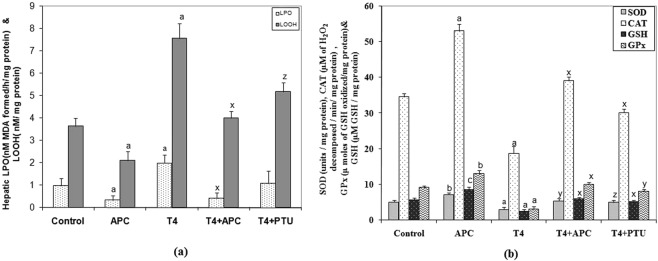
(**a**) Changes in LPO **(**nM MDA formed/h/mg protein) and lipid hydroperoxides (LOOH, nM/mg protein) in the hepatic tissues of APC (2.0 mg/kg/d) alone or T_4_ + APC or T_4_ + PTU treated animals. Each vertical bar represents the mean ± SEM. (n = 7). ^a^*P* < 0.001 and ^c^*P* < 0.05 as compared to the respective control value, whereas ^x^*P* < 0.001 as compared to the L-T_4_ treated animals. (**b**) Activities of SOD (units/mg protein, CAT (µM of H_2_O_2_ decomposed/minute/mg protein), GPx (μ moles of GSH oxidized/mg protein) and GSH (μMGSH/mg protein) in liver tissues following the administration of either T_4_ alone or with APC. Each vertical bar represents the mean ± SEM (n = 7). ^a^*P* < 0.001, ^b^*P* < 0.01 and ^c^*P* < 0.05 as compared to the respective control value. ^x^*P* < 0.001, ^y^*P* < 0.01 and ^z^*P* < 0.05 as compared to the respective value of the T_4_-induced animals.

In T_4_-induced rats, the levels of lipid peroxidation (LPO) products such as thiobarbituric acid reactive substances (TBARS) and lipid hydroperoxides (LOOH) were significantly high as compared to control rats. However, APC administration to thyrotoxic rats or to euthyroid animals significantly reduced the levels of LPO (Fig. 4a). In APC alone treated animals also a significant decrease in LPO and LOOH was observed as compared to that of control animals.

While the activities of antioxidants such as superoxide dismutase (SOD), catalase (CAT), glutathione peroxidase (GPx) and total glutathione (GSH) content were depleted significantly in the liver of T_4_-treated animals, following the administration of APC, the activities of these antioxidants were restored near to control levels. In T_4_ + PTU treated groups the values were also more or less similar to that of control (Fig. 4b). Administration of APC alone too increased the antioxidants to a significant level as compared to that of control animals.

### Western blot analyses

**Figure 5:**
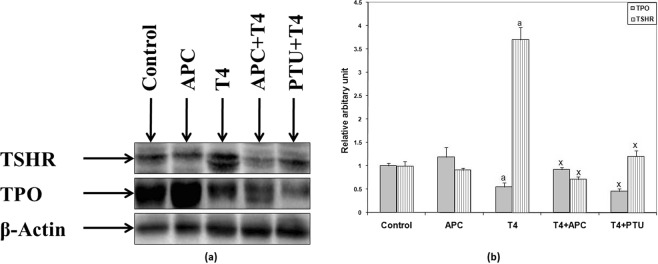
(**a**) Results of western blotting on the protein expression of TPO and TSHR in the thyroid gland showing the effects of APC in euthyroid and thyrotoxic animals. (**b**) Representative western blot of TPO and TSHR quantification showing the down regulation of TPO and high expression of TSHR in the thyroid gland of T_4_-induced thyrotoxic rats. In T_4_ + APC and T_4_ + PTU treated animals, TPO expression was upregulated and TSHR was down regulated. ^a^*P* < 0.001 as compared to the control value and ^x^*P* < 0.001, as compared to the value of the T_4_- induced animals. Data were analyzed by one way analysis of variance (ANOVA) followed by Tukey–Kramer post-hoc test and have been expressed as the mean ± SEM (n = 5 per each group).

In the expression of thyroid peroxidase (TPO) and thyroid stimulating hormone receptor (TSHR) marked changes were observed in thyrotoxic rats. TPO expression was significantly down-regulated, while TSHR expression got upregulated. However, treatment of APC or PTU to thyrotoxic rats significantly increased the expression of TPO and decreased the TSHR (Fig. 5a). On quantification of the expressions of TPO and TSHR in thyroid tissues (Fig. 5b), in thyrotoxic animals, a significant decrease in the level of TPO, but an increase in TSHR was observed as compared to their respective control values. However, in APC + T_4_ and in PTU + T_4_ treated rats, TPO was markedly increased as compared to the value of thyrotoxic animals. Full-length gel has been presented in Supplementary Section (Fig. S2a,b).

### Histological changes in the liver and thyroid tissues

**Figure 6:**
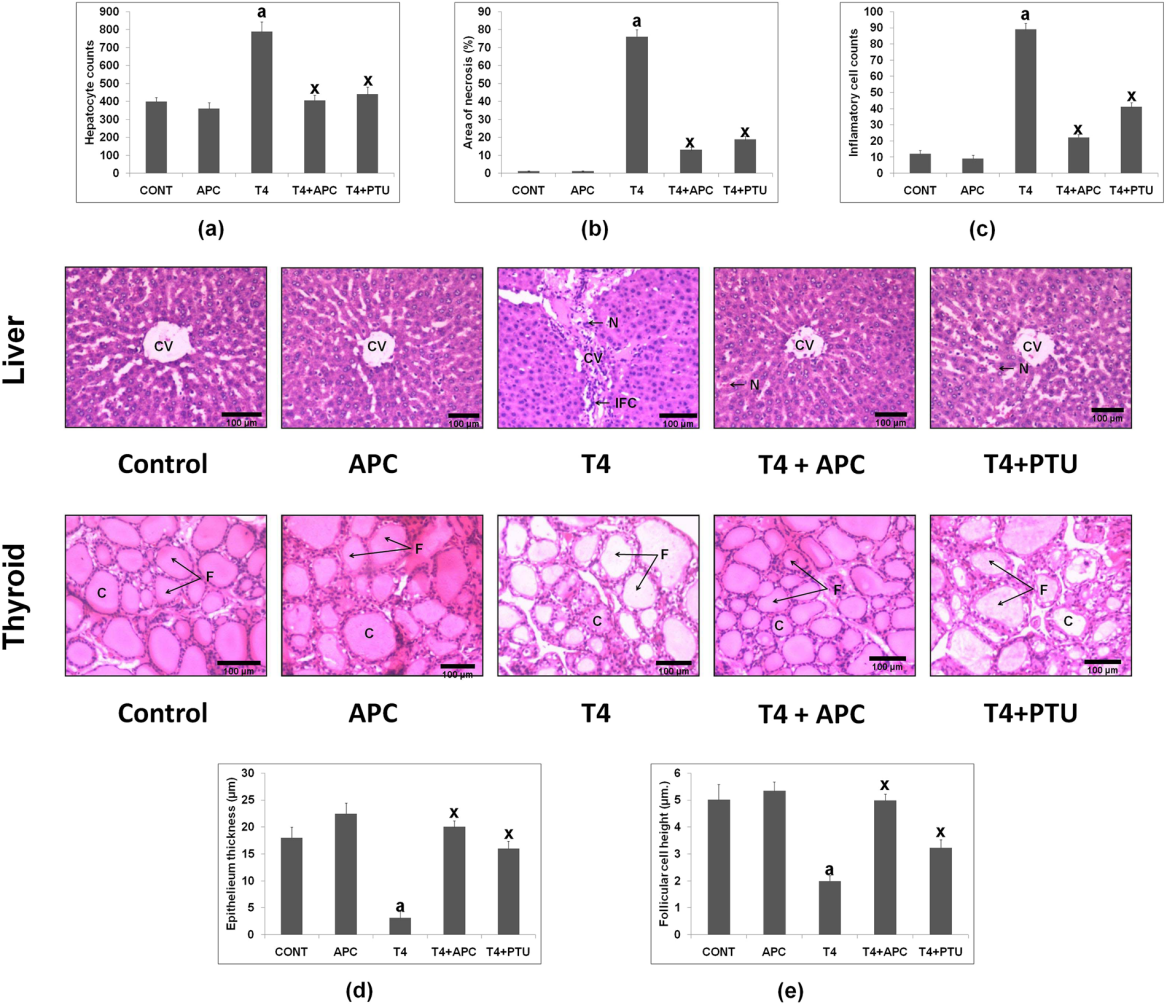
Effects of APC on the histological changes in liver and thyroid gland of T_4_ treated rats [H and E stain (10x)]. Liver section of control group shows the central vein (CV) surrounded by the radiating cords of hepatocytes, whereas T_4_-induced rat liver shows inflammatory cell infiltrations and cellular damage. In liver section of APC treated animal, cords of normal hepatocytes (H) radiating from the central vein (CV) are seen. In T_4_-induced APC treated rat liver, near normal histological structure is seen with normal hepatocytes. Only moderate degree of liver damage is seen in the T_4_-induced animals that received PTU also. N = necrotic area, IFC = inflammatory cells (arrows). (**a**) In T_4_-induced thyrotoxic liver, an increase in the hepatocytes number was found, but in T_4_ + APC and T_4_ + PTU groups, there was a reduction in the number of hepatocytes as compared to that of thyrotoxic liver. Data are presented as mean ± SEM (n = 5 per each group). ^a^*P* < 0.001 as compared to the respective control value. ^x^*P* < 0.001 as compared to the respective value of the T_4_-induced animals. (**b**) Quantitative analysis of liver necrosis. In T_4_- treated rats the percent necrotic area was increased as compared to that of control rats (n = 5). This was determined by random evaluation of each HE-stained section. However, in T_4_ + APC and T_4_ + PTU groups, decreased area of necrosis (%) was observed as compared to that of thyrotoxic liver. Data are presented as mean ± SEM. (n = 5 in each group). ^a^*P* < 0.001 as compared to the respective control value. ^x^*P* < 0.001 as compared to the respective value of the T_4_- induced animals. (**c**) Histological changes also indicate increased inflammatory cell count in T_4_-induced thyrotoxic liver which is reduced in T_4_ + APC and T_4_ + PTU treated animals, as compared to the thyrotoxic liver. Data are presented as mean ± SEM. (n = 5 in each group). ^a^*P* < 0.001 as compared to the respective control value. ^x^*P* < 0.001 as compared to the respective value of the T_4_- induced animals. Representative photomicrographs of thyroid tissues in H&E-stained images (10x) showing marked changes in thyroid section of APC or T_4_ + APC or PTU treated rats. In control rats normal thyroid follicles (F) lined with a single layer of cuboidal follicular cells are seen, while in T_4_–induced rats, thyroid follicles are markedly distended with less amount of colloid. The thyroid sections of animals treated with T_4_ + APC or T_4_ + PTU show marked improvement in the thyroid follicles. In only APC treated animal, no alteration in the histological structure of thyroid follicles is seen exhibiting nearly normal histological features. F = follicle; C = colloid. (**d**) Changes in the follicular lining epithelium thickness (measured as morphological assessment) of the thyroid gland are seen following APC treatment. While epithelium thickness was reduced in T_4_-induced thyrotoxic rats, thyroid section of T_4_ + APC and T_4_ + PTU showed almost normal follicular lining epithelium. Data are presented as mean ± SEM (n = 5 per group). ^a^*P* < 0.001 as compared to the respective control value, whereas ^x^*P* < 0.001, as compared to the respective value of thyroxin treated animals. **(e**) It shows a reduction in the follicular cell height in T_4_-induced thyrotoxic rats, but thyroid section of animal, treated with T_4_ + APC and T_4_ + PTU show normal follicular cell height. Data are presented as mean ± SEM (n = 5 per group). ^a^*P* < 0.001 as compared to the respective control value, whereas ^x^*P* < 0.001, as compared to the respective value of thyroxin treated animals.

The liver of control rat showed a normal histological structure with cords of polyhedral hepatocytes radiating from the central vein, while T_4_-induced liver indicated severe hepatic damage with disorganization of hepatic cords, the central vein and release of cellular inflammatory cells in portal tract. However, administration of APC to L-T_4_-treated rats improved the liver architecture to more or less similar to that of control group by preventing the cellular damage and inflammation As shown in Fig. 6(a), the hepatocytes count in thyrotoxic group was significantly more as compared to control animals. T_4_-induced rats resulted an increase in necrotic area as compared to that of control animals whereas, APC or PTU treatment reduced the necrotic area significantly as compared to that of T_4_-induced rats (Fig. 6b). In thyrotoxic rats, a significant increase in the number of inflammatory cell counts was observed as compared to that of control. However, the liver sections of T_4_ + APC and T_4_ + PTU treated rats showed a reduction in inflammatory cell count with respect to hyperthyroid rats (Fig. 6c).

Thyroid gland histology (Fig. 6) from the control and APC alone treated animals exhibited normal histological architecture with multiple follicles consisting of a layer of follicular cells and filled with moderate amount of colloid. However, examination of thyroid gland sections of T_4_-induced rat exhibited decreased thickness of follicular lining of epithelium (Fig. 6d) and decreased follicular cell height (Fig. 6e). Following the treatment with APC or PTU to thyrotoxic animals the follicular sizes and follicular lining of epithelium were nearly normalized.

## Discussion

The isolated compound from betel leaf was identified as allylpyrocatechol with a molecular formula, C_9_H_10_O_2_, deduced on the basis of its spectroscopic analyses (IR, ^1^H, ^13^C NMR and GC-MS). Although, this compound was isolated earlier from piper betel plant, investigation on its biological effects were primarily done with respect to antioxidative, gastro-protective and anti-inflammatory properties only^11,12,14^ and practically nothing was studied in relation to the regulation of thyroid dysfunction. Findings of this *in vivo* study clearly indicated the ameliorative nature of the test compound, APC in T_4_-induced hyperthyroid rats, suggesting its therapeutic use in thyrotoxicosis.

The chronic administration of L-T_4_ induced thyrotoxicosis in rats as evidenced by marked increase in the level of serum thyroid hormones and in the activity of hepatic 5′D1 (the enzyme, that converts T_4_ to T_3_), with a decrease in TSH. This was also supported by loss of body weight, which may be due to the increase in the body metabolism by excess amount of circulatory thyroid hormones^22^. However, when APC was administered to T_4_-induced animals, it decreased the levels of both T_4_ and T_3_ and increased the serum TSH, suggesting an inhibition in thyroid hormone synthesis and/or their release. Interestingly, hepatic 5′-D1 activity was also inhibited by the test compound, ascertaining that APC has the potential not only to inhibit the glandular synthesis and/or secretion of T_4_, but also the peripheral conversion of T_4_ to T_3_, the major pathway of production of latter thyroid hormone. These findings are somewhat similar to the earlier observations with another bioactive compound that exhibited thyroid-inhibitory action in rats^10^.

As thyroid hormone synthesis was inhibited by the test drug, attempt was made to reveal its action on the process of iodination of tyrosine molecule, the first step of thyroid hormone synthesis, in which TPO plays an important role^23^, i.e., oxidation of inorganic iodide (I^−^) to reactive iodine (I^0^) for its binding to tyrosine molecule, the main amino acid of the thyroid hormones. In another animal model, less or negligible TPO expression was seen in the absence of TSH/TSHR signalling, indicating that TPO expression is regulated largely by TSH^24^. Interestingly, in our experimental thyrotoxic rats, not only there was less TSH, but also the expression of TPO was down regulated. However, following the APC treatment in thyrotoxic animals, a significant increase in both the indices was observed. These findings may be compared with an earlier report, in which anti-thyroid compounds such as methimazole and propylthiouracil increased cellular thyroid peroxidase activity in cultured porcine thyroid follicles^25^. Moreover, as high TSH level is known to increase the level of TPO antibody^26^, it is quite possible that APC-induced increase in TSH might have directly increased the TPO expression in this investigation.

Thyroid-stimulating hormone and thyrotropin receptor are directly involved in the regulation of thyroid function^27^. In fact, TSHR plays a pivotal role in thyroid hormone metabolism. Since this receptor responds to TSH and stimulates the production of thyroid hormones, it was suggested earlier that in Graves disease (a condition of hyperthyroidism), the activation of TSHR is seen with low or no TSH^28,29^. We also observed a higher expression of TSHR protein and lower expression of TPO in L-T_4_-induced thyrotoxic rats that had low TSH level. However, following APC treatment in thyrotoxic animals, there was a marked decrease in TSHR expression and an increase in TPO expression as compared to T_4_-induced rats, confirming that APC suppressed the thyroid function.

Thyroid hormones normally promote metabolic conditions, characterized by weight loss, reduced levels of different serum lipids including cholesterol, TG and HDL^30,31^. In this study, similar to our earlier finding^10^ decreased levels of TC, TG, HDL-C, LDL-C, and VLDL-C were observed in thyrotoxic rats; whereas, treatment with APC restored all these serum lipids near to their normal levels in T_4_-treated rats, further supporting its beneficial effects in thyrotoxicosis.

G-6-pase is often considered as an important enzyme in thyroid function and its activity is closely related to the thyroidal activity or to the circulating levels of thyroid hormones^32,33^. Interestingly, we also found that the activity of this enzyme was increased by T_4_, but decreased in T_4_ -induced APC treated animals, again consolidating its potential to regulate thyrotoxicosis.

An increase in hepatic Na^+^/K^+^-ATPase activity was noticed in the thyrotoxic animals, as reported earlier^34^. However, treatment with APC decreased the same, further supporting the thyro-inhibitory role of APC. This effect of APC was similar to that of PTU in thyrotoxic rats.

An increase in reactive oxygen species induced by L-T_4_ always leads to oxidative stress in different tissues including liver, the major target organ of a drug, with consequent lipid peroxidative responses^35,36^. In this study also, thyrotoxicosis resulted in a marked increase in malondialdehyde (MDA) and lipid hyroperoxides(LOOH) levels with a depletion of antioxidants. Interestingly, following the administration APC in L-T_4_ -induced rats, not only tissue LPO was reduced, but also it increased the activities of antioxidants such as SOD, CAT, and GPx, which provide the first line of defence against superoxide and hydrogen peroxides^37^.

As reported previously, we also found low levels of hepatic GSH content in T_4_ -induced animals^38,39^. Interestingly, following the treatment with APC to thyrotoxic rats, GSH level was normalized. Thus APC could increase the antioxidant levels in liver clearly indicating its efficacy on decreasing oxidative stress provoked by thyrotoxicosis. This may be emphasized that the test compound exhibited better antioxidative effects as compared to that of PTU.

ALT, AST and LDH are considered as sensitive indicators of liver cell injury and their high levels are often associated with hepatic dysfunction^40^. We also observed higher activities of these enzymes in serum of thyrotoxic rats, in response to oxidative stress, induced by Thyrotoxicosis as observed earlier in another study^41^. However, following APC treatment, ALT, AST and LDH levels were reduced thus cellular and tissue damage was prevented by APC. This tissue protective effects of APC is in accordance with an earlier report^42^. While studying the cytokine levels an increase in serum TNF-α was found in thyrotoxic animals as compared to the value of control animals. TNF-α has an effective impact on the circulating antibody production during inflammation^43^. In fact, an increased oxidative stress is known to stimulate the release of different cytokines, including TNF-*α* and interleukins^44^. This might explain the high levels of TNF-*α* observed in the thyrotoxic group. Since the levels of TNF-*α* was significantly lower in rats receiving APC, as compared to the value of thyrotoxic rats, it appears that APC might have a potent anti-inflammatory activity, at least in L-T_4_-induced thyrotoxic rats.

In the liver of thyrotoxic animals, we found an increase in hepatocyte number as reported in an earlier finding^45^. However, treatment with APC restored the normal hepatocytes count. Further, the inflammatory cells count was increased in thyrotoxic liver and less inflammatory cell counts were seen following APC administration which could be the result of consequent inhibition of the production of TNF-*α.* The hepatic damage, in terms of necrotic area due to excess thyroid hormones was also observed. This may be related to enhanced lipid peroxidation and higher levels of ALT, AST, LDH and TNF-α in T_4_ –induced rats. Interestingly, the treatment with APC resulted in a marked decrease in necrotic area when compared to that of thyrotoxic group, suggesting the hepato-protective effects through attenuation of oxidation and inflammatory response in the liver by APC.

With respect to thyroidal histology, while normal thyroid structure with cuboidal to low columnar, small and medium-sized follicles were seen in euthyroid control animals, following L-T_4_ administration, marked distortion in its architecture was observed. Similar to the earlier studies^46,47^, we also found a reduction in the height and epithelial thickness of the follicles and hypertrophied follicles with less amount of colloid in L-T_4_ induced rats, which were nearly corrected by APC treatment. APC also decreased T_4_ and T_3_ levels and increased the TSH concentration, exhibiting anti thyroidal property of the test compound.

In fact, the effects of the APC on thyroidal histology were comparable with that of PTU. An important point that needs to be emphasized here is that the dose of APC that we used *in vivo* is equivalent to a dose of about 130 mg/kg/day in human, according to dose translation from animal to human^48^.

At present, nothing can be said on the exact molecular mechanism(s) of APC effects. One explanation could be that, the inhibition of thyroid functions by the test compound could be the result of decrease in T_4_ synthesis and/or secretion at the glandular level and by the reduction of T_4_ to T_3_ conversion, an important process of T_3_ formation. Another key mechanism is that, APC might have ameliorated the thyrotoxicosis by regulating the expression of TPO and TSHR proteins.

In conclusion, the isolated compound allylpyrocatechol exhibited its potential in minimizing the pathophysiology of T_4_-induced thyrotoxicosis in rats with additional hepatoprotective effects. Possibly this anti-thyroid action of APC is due to the inhibition of synthesis and/or release of T_4_ and by a decrease in the T_3_ production, primarily by altering the 5′D1 activity in liver.

On the cellular mechanism of action of the test compound, it seems that APC increased and decreased the expression of thyroidal TPO and TSHR respectively. This may be a novel finding that suggests a beneficial role of our test compound, APC in the amelioration of thyrotoxicosis. Of course, further investigation is required to establish the optimum dose for its therapeutic use.

### Limitation

The limitation in this experiment is that we did not study the m-RNA expression and its relationship with protein levels. As the changes in gene expression may not always support the changes in m-RNA expression, we preferred to carry-out only protein expression studies to avoid the uncertainty on the effects of APC in thyroid function.

## Materials and Methods

### Chemicals and assay kits

Enzyme-linked immunosorbent assay (ELISA) kits for thyroid hormone assays were purchased from Life Technologies Pvt. Ltd., India. For estimations of total cholesterol, triglyceride and high density lipoprotein cholesterol, assay kits were obtained from Span diagnostics Pvt. Ltd., Surat, India. Kits for aminotransferase enzymes, alanine transaminase, aspartate transaminase, and lactate dehydrogenase were from Erba diagnostic pvt. Ltd., GmbH, Germany. ELISA kit for TNF-α was purchased from Ray Biotech Inc, Norcross, GA, USA. While anti-TPO, anti-TSHR, anti-β-actin and nitrocellulose were obtained from Santa Cruz Biotechnology, USA; NC membrane was supplied by Millipore, USA. Jackson Immuno-Research Laboratories Inc. USA supplied HRP-conjugated anti-mouse secondary antibody. Bovine serum albumin (BSA) and Tween-20 were provided by Sigma-Aldrich, USA. All other routine chemicals used in the biochemical studies were purchased from Hi-Media, Mumbai, India.

### Plant material

*P*. *betel* leaves of Mysore variety were purchased from local market, Indore, India. The test plant material was authenticated by a known botanist Prof. A. Serwani and deposited with the voucher number, BL 06102 in School of Life Sciences, Devi Ahilya University, Indore, India.

### Extraction and isolation

Ethanol extract of the *P*. *betel* was prepared from its dry leaf powder and then APC was isolated from it using the procedure reported earlier^21^. Dried powder of *P*. *betel leaf* (250 g) was extracted with 95% ethanol (1 L) for 2 days. The extract was filtered and the supernatant was collected. The process was repeated thrice and all the clear fractions were pooled and evaporated in vacuum. Eight gram of this extract was dissolved in 50 mL of methanol, treated with 0.2 g of activated charcoal and warmed at 60 °C. The insoluble material was removed by filtration and the extract was dried under vacuum and lyophilized to afford the chlorophyll free amorphous yellowish brown solid (yield, 0.98%). This was stored in a vacuum desiccator. The ethanol extract (4.7 g) was chromatographed over a silica gel column and 50 mL of fractions were collected using the gradient elution of 0, 2, 5 and 10% ethyl acetate/hexane followed by 0, 5, 10, 15, 50 and 100% methanol/chloroform (500 mL each). The fraction eluted with 10% ethyl acetate/hexane yielded the compound APC as light yellow oil and the yield was 0.92% (w/w of the extract). The structure of the isolated APC (Fig. 1) was elucidated using different spectral analyses such as IR, NMR and GC/MS.

### Spectral data analyses

Gas chromatography mass spectroscopy (GC-MS) analysis of the isolated compound was carried out on JEOL GCMATE II system from Agilent Technologies, USA. The HP-5 column used in GC was of 30 m long and 0.25 mm diameter. Purity of APC was determined by HPLC using a Shimadzu LC-20AT system equipped with Shimadzu diode array detector. For chromatographic separation, C18 column (4.6 mm × 250 mm, and particle size 5 µm), from Agilent Technologies Inc., Santa Clara, CA, USA was employed. The ^**1**^H and ^13^C NMR spectra were recorded on Bruker NMR spectrometer at 500 and 125 MHz respectively in CDCl_3_ as solvent and tetramethylsilane (TMS) as the internal standard. Chemical shifts are expressed in δ (ppm).

#### Animal ethics

Handling of animals, their maintenance and treatment with drugs in animals were conducted according to the guidelines of the Ministry of Social Justice and Empowerment, Government of India (registration No, 779/Po/Ere/S/03/CPCSEA). The experimental procedure was approved by our Institutional Animal Ethical Committee (IAEC) and the Departmental Ethical Committee (School of Life Science, DAVV, Indore, India).

### Experimental design

In our first experiment, an initial screening of three different concentrations (0.5, 1.0, and 2.0 mg/kg, suspended in 1% acacia gum) of the test compound was made to find out the most effective dose of APC in L-T_4_ treated (pre standardized 500 µg/kg of L-T_4_ for 12 days) female thyrotoxic rats. We observed that both T_3_ and T_4_ levels were significantly inhibited at 2.0 mg/kg without any hepatotoxic effects (data shown in Supplementary Section, Table 1). Based on these results, the effective dose, 2.0 mg/kg was selected for the next experiment.

In the second experiment, healthy female Wister rats, weighing 170 ± 10 g were divided in to five groups and in each group seven animals were kept. The control animals (group 1) received gum acacia in distilled water (per oral, p.o); Group 2, 4 and 5 were first injected with 500 µg/kg of L-T_4_ for 12 days to induce thyrotoxicosis. While T_4_-induced group 4 animals were treated with APC (2.0 mg/kg, p.o.), animals of group 5 received PTU (10 mg/kg, i.p.) and animals of group 3 received only APC by oral route for 30 days. The doses here we used for PTU and T_4_ were taken from our previous published work^31^. To avoid the circadian interference, all the treatments were scheduled between 10.00 and 11.00 h of the day and the total duration of the experiment was 6 weeks. After 6 weeks, the overnight fasted rats were anesthetized and sacrificed by cervical decapitation. Blood was collected, allowed to clot and serum was separated by centrifugation. Immediately the liver and thyroid glands were removed and washed with cold saline. Liver tissue homogenate (10%, w/v) was prepared in ice-cold phosphate buffered saline (0.1 M, pH 7.4) and centrifuged for 20 min at 10,000 × g and at 4 °C. The supernatant was used for the assay of different biochemical parameters.

### Assay of serum thyroid hormones, TSH and TNF-α

T_3_, T_4_ and TSH were estimated in serum by ELISA using the commercial kit from Life Technologies (India) Pvt. Ltd. following the manufacturer’s instructions. The levels of TNF-α in serum was estimated using the commercially available specific ELISA kit.

### Hepatic 5′D1 activity

The activity of 5′D1 was measured in liver homogenates with the protocol used earlier by Kodding *et al*.^49^ with little alteration. Liver of each animal was homogenized in ice-cold phosphate buffer (0.15 M, pH 7.2) with 0.25 m sucrose and 5 mM EDTA. The homogenates were centrifuged at 2000 g for 30 min at 4 °C and the supernatants were collected. To100 μl of supernatants, 10 μl of T_4_ (4 µM) and 10 μl of dithiothreitol (DTT) (4 mM) were added and kept for incubation at 37 °C for one hour. The reaction was stopped by the addition of 800 μl of absolute ethanol. The amount of T_3_ formed was measured using the T_3_ ELISA kit.

### ALT, AST and LDH activities

Enzyme activities of ALT, AST and LDH were estimated in serum according to the manufacturer’s instructions mentioned in the specific kit.

### Concentration of serum lipids

For the estimation of **s**erum TC, TG and HDL-C, commercial kits were used. LDL-C and VLDL-C values were calculated out, for which the formula of Friedwald *et al*.^50^ was used. The formula is, LDL-C = Total cholesterol − (HDL-C + VLDL-C) and VLDL-C = Triglycerides/5.

### Assay of Glucose-6-phosphatase

The activity of this enzyme was assayed by the method Bagniski *et al*.^51^. For this we measured the amount of inorganic phosphate (Pi) released from an incubation reaction (for 15 min and at 30 °C) mixture consisting of liver supernatant (0.3 mL), 0.7 mL of citrate buffer (0.1 M, pH 6.5) and 0.3 mL of glucose-6-phosphatase (20 mM). The reaction was stopped by the addition of 1 mL of 10% TCA, and then centrifuged to obtain the clear supernatant which was processed for the estimation of Pi by the method of Fiske and Subbarow^52^. Absorbance was taken at 680 nm.

### Assay of Na^+^-K^+^-ATPase

The hepatic Na ^+^ -K^+^-ATPase activity was determined by the method of Esmann *et al*.^53^ in which the liberated inorganic phosphate was measured by the method of Fiske and Subbarow^52^. The enzyme activity was expressed in nmol of Pi liberated/hr/mg protein. The routine method of Lowry *et al*.^54^ was used for the estimation of tissue protein.

### Study of oxidative stress markers: LPO and LOOH

In liver tissue LPO was studied by measuring the amount of thiobarbituric acid reactive substances (TBARS) using the method of Ohkawa *et al*.^55^. To the liver homogenate (0.2 mL), the reaction mixture consisting of 0.2 mL of 8.1% sodium dodecyl sulfate (SDS), 1.5 mL of 20% acetic acid (pH3.5) and 1.5 mL of 0.8% of thiobarbituric acid (TBA) were added, followed by heating for 1 hr at 95 °C and cooled. To this, 5 mL of n-butanol and pyridine mixture (15:1 v/v) were added, centrifuged at 5000 rpm for 20 min and the absorbance of organic layer was measured at 532 nm. The unit for expression of LPO was nM MDA formed/h/mg protein.

For measuring lipid hydroperoxides levels, the method of Jiang *et al*.^56^ was followed. In brief, to 0.1 mL of liver supernatant, 0.9 mL of Fox reagent was added and incubated for 30 min in room temperature, centrifuged and the absorbance of upper layer was taken at 560 nm. It was finally expressed in nM of LOOH formed/mg protein.

### Assessment of antioxidants

#### Superoxide dismutase (SOD)

The SOD activity in hepatic tissue was estimated using the method of Marklund and Marklund^57^. In brief, to 20 µl of liver homogenate, 2 mL of 50 mM Tris-HCl buffer (pH 8.2) and 1 mL (2 mM) pyragallol were added and absorbance was measured at 420 nm for 3 min. Inhibition of the rate of pyragallol autooxidation by 50% is considered as one unit of the enzyme activity.

### Catalase activity

Catalase (CAT) activity in liver was estimated according to the method of Aebi^58^. Liver homogenate (50 µl) was added to 1.0 mL of 50 mM phosphate buffer (pH 7). Then the reaction was initiated by the addition of 1 mL of 30 mM H_2_O_2_. The decrease in absorbance due to H_2_O_2_ decomposition was read at 240 nm for 3 min at the interval of 60 sec and the enzyme activity was expressed in μM of H_2_O_2_ decomposed/min/mg protein.

### Estimation of reduced glutathione (GSH)

The amount of GSH level was measured by the method of Ellman^59^. Liver supernatant (0.5 mL) was first precipitated with 2.0 mL of 5% TCA., centrifuged and the clear supernatant (1.0 mL) was added with 0.5 mL of Ellman’s reagent and 3.0 mL of phosphate buffer. GSH reaction with DTNB produced a yellow-colored complex (2-nitro-5-mercaptobenzoic acid) whose absorbance was read at 412 nm.

### Glutathione peroxidase(GPx) assay

The assay procedure of Rotruck *et al*.^60^ was followed for the estimation of GPx. To the reaction mixture (0.2 mL of 0.4 M tris buffer at pH 7.0, 0.2 mL of ethylene diamine tetracetic acid, 0.1 mL of sodium azide), 0.5 mL of liver homogenate was added. To this mixture, further 0.2 mL of glutathione and 0.1 mL of H_2_O_2_ were added and incubated at 37 °C for 10 min. To stop the reaction. 0.5 mL of 10% TCA was added, centrifuged and the supernatant was processed for the estimation of glutathione. GPx activity was expressed in μg of GSH consumed/min/mg protein.

### Western blotting

As carried out earlier^61^, individual protein samples (20 μg each) extracted from thyroid gland were separated on a 12% SDS-polyacrylamide gel and then transferred on to a nitrocellulose membrane electrophoretically. The membrane was blocked in a 5% BSA and 0.5% Tween-20 for 1 h with mild shaking at room temperature followed by an overnight incubation at 4 °C with the primary antibodies, anti-TPO (1:2000 dilutions), anti-TSHR (1:2000 dilutions) and internal loading control β-actin (1:2000 dilutions) overnight at 4 °C. After washing thrice with sterile phosphate buffer saline (PBS) and 0.05% Tween-20, incubated with horseradish peroxidase (HRP) -conjugated secondary anti-mouse antibody (1:5000 dilutions) for 1 h at room temperature. The membrane was then developed using enhanced chemiluminescence (ECL), Thermo Scientific, Pierce, USA, and visualized using ChemiDoc^TM^ MP Imaging system (Bio-Rad, USA). Western blot images were quantified using image Lab^TM^ software version 5.1 (Bio-Rad).

### Histological analyses of thyroid and liver tissues

Liver and thyroid tissues were fixed in 10% neutral formalin and embedded in paraffin. The sections were cut into (5-μm thickness), stained with hematoxylin-eosin (H & E) and were observed under light microscopy (10x magnification). Quantification of morphometric changes of thyroid follicular cell height, thyroid epithelium thickness and hepatocytes count has been performed by ImageJ NIH analysis software, as done earlier by Hwang *et al*.^62^.

### Statistical analysis

All values were expressed as mean ± SEM. Results were statistically analysed using the Graph-Pad Instat software, by one-way analysis of variance (ANOVA) followed by post-hoc Tukey–Kramer multiple comparison test. A level of *P* < 0.05 was considered statistically significant. Only T_4_-induced rats and APC alone treated rats were compared with normal rats and APC, PTU treated rats were compared with T_4_-treated animals.

## Supplementary information

Allylpyrocatechol, isolated from betel leaf ameliorates thyrotoxicosis in rats by altering thyroid peroxidase and thyrotropin receptors
