# Supplementary material for: Allylpyrocatechol, isolated from betel leaf ameliorates thyrotoxicosis in rats by altering thyroid peroxidase and thyrotropin receptors
Source: Sci Rep. 2019 Aug 22;9:12276. doi: 10.1038/s41598-019-48653-9 (PMC6706422; doi:10.1038/s41598-019-48653-9)
Supplement: Supplementary file 1 — Allylpyrocatechol, isolated from betel leaf ameliorates thyrotoxicosis in rats by altering thyroid peroxidase and thyrotropin receptors [file 41598_2019_48653_MOESM1_ESM.pdf]

Allylpyrocatechol, isolated from betel leaf ameliorates thyrotoxicosis in rats by altering thyroid peroxidase and thyrotropin receptors

Sunanda Panda[1*], Malabika Sikdar[2], Sagarika Biswas[3], Rajesh Sharma[1], Anand Kar [4]

[1]School of Pharmacy, , Devi Ahilya University, Indore, India.[2] Department of Zoology, Dr. Hari Singh Gour Vishwavidyalaya, Saugar, India; [3]Department of Genomics and Molecular Medicine, CSIR-Institute of Genomics and Integrative Biology, New Delhi, India and [4] Thyroid Research lab, School of Life Sciences, Devi Ahilya University, Indore, India.

Supplementary / supporting information on isolated APC

The High-performance liquid chromatography (HPLC) system comprised of a Shimadzu LC - 20AT system equipped with a SPD-M20A diode array detector and the column used for this experiment was C18 column (4.6 × 250 nm, 5 µm), Agilent Technologies Inc., Santa Clara, CA, USA). A solution of APC (100 µg/ml)) in acetonitrile was prepared .The injection volume was of 10µl and the sample was analyzed by isocratic method using the mobile phase acetonitrile: water (1:1, v/v, + 0.02% acetic acid) at a , flow rate of 1 mL/min for 30 min. Photodiode array detector setting was at 254 nm for obtaining the chromatograms. The purity of APC was determined as 98.6% by peak area normalization method .The important parameters, including retention time and wavelength with maximum absorbance (254 nm), of the isolated APC peak was the same as that of the APC standard (5.07 min), as shown in Figure (Fig. 1b &1c).

GC-MS analyses of isolated APC

The GC-MS analysis of APC was performed using JEOL GCMATE II GC-MS (Agilent Technologies 6890N, Network GC system for gas chromatography). The column (HP5) was fused with silica 50 m x 0.25 mm I.D. For GC-MS detection, an electron ionization system with ionization energy of 70 eV was used. The mass range was 60 to 170 m/z ; GC conditions: Initial oven temperature 70°C, hold 2.0 min to 305°C at 20°C/min and hold for 1 min. Helium gas used as carrier gas, the ion source temperature set at 230°C. Total running time 20 min. flow rate ,1.2 ml/min. The identification of compound was done by comparing the mass spectra of the respective peaks obtained in the GC-MS with the mass fragmentation patterns of standards, in the National Institute of Standard Technology (NIST MS) search library Software version 2.0.

Spectral data analyses

IR spectra were scanned as thin films within Perkin Elmer system, FT-IR/ATR and the ^{1}H NMR and ^{13}C NMR spectra were obtained using Bruker DRX-500 (500 MHz FT- NMR) spectrometer in $CDCl_3$. Tetramethyl saline was used as internal standard. Chemical shifts were expressed in δ values.

Preliminary experiment on dose standardization of APC(data given below and explained in main text).

Table 1
Effects of 0.5, 1.0 and 2.0 mg/kg of APC in hepatic LPO (nM MDA formed/ h/mg protein) and in serum $T_3$and T_4 concentration (ng /ml for both) in rats.

Groups	LPO	T_3	T_4
Cont.	0.54 ±0.03	0.65 ±0.04	52.77 ±2.34
T_4	1.38[a] ± 0.06	2.01[a] ±0.86	139.16[a] ± 5.02
T_4 + APC, 0.5 mg/kg	1.01 ± 0.06	1.68 ± 0.028	110.6[z] ±7.84
T_4+ APC,1.0 mg/kg	0.77[x] ±0.03	0.99 [x] ±0.041	96.82[x] ±4.99
T_4+ APC, 2.0 mg/kg	0.34[x] ±0.02	0.29 [x] ±0.03	43.99 [x] ± 3.82

Values are expressed as mean ±SEM; n =7. [a] $P<0.001$ as compared to the respective control values.. [x] $P<0.001$ as compared to the respective value of T_4- treated group.

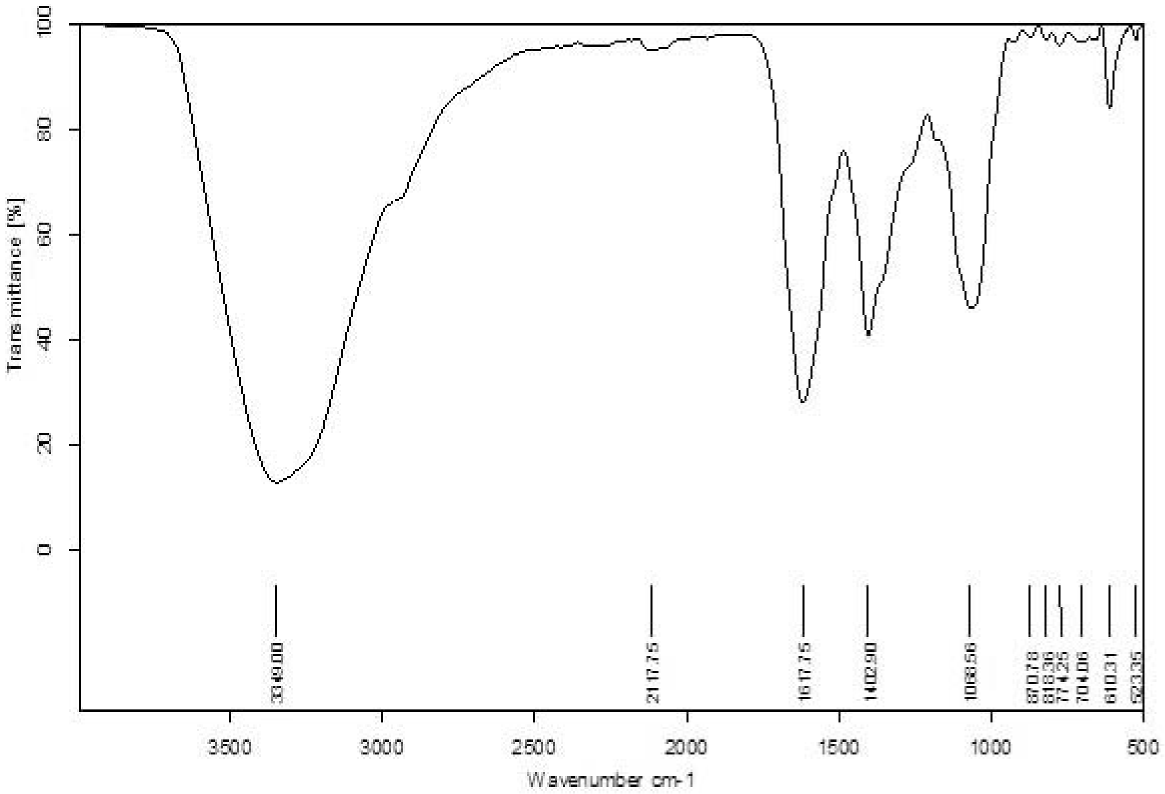

Figure S1 a. Infrared (IR) spectra data of APC.

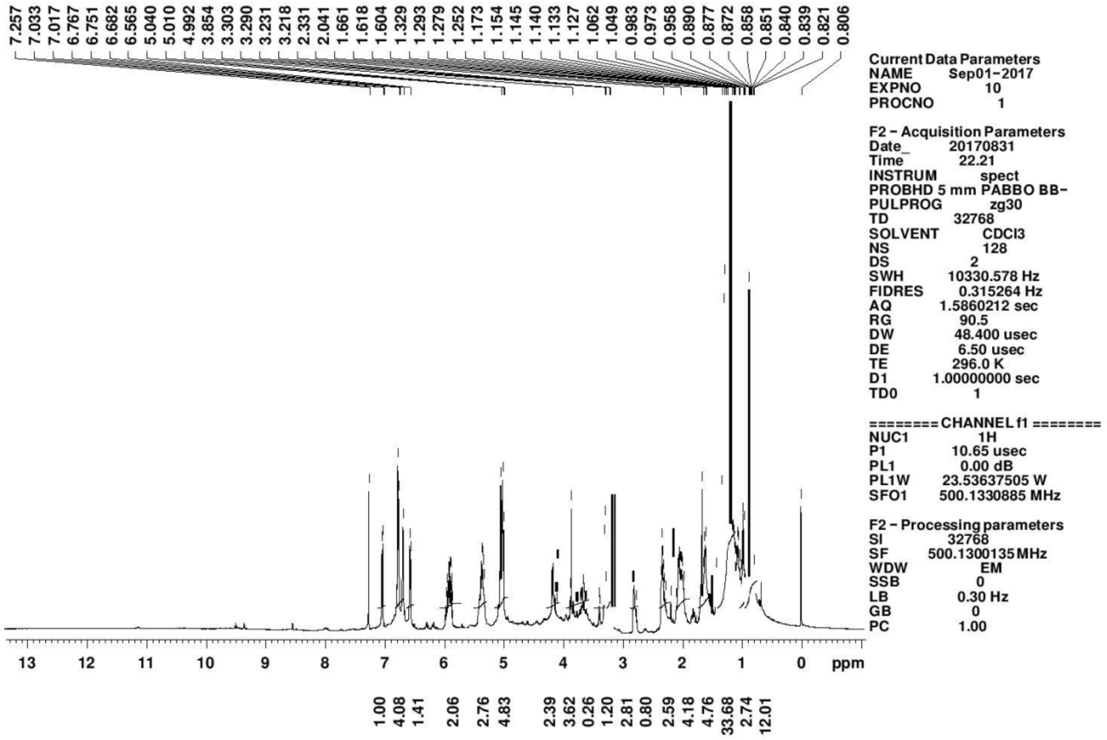

Figure S1b. Proton nuclear magnetic resonance (^{1}H NMR, 500 MHz; δ in ppm, J in Hz) spectrum data of APC. Recorded in $CDCl_3$.

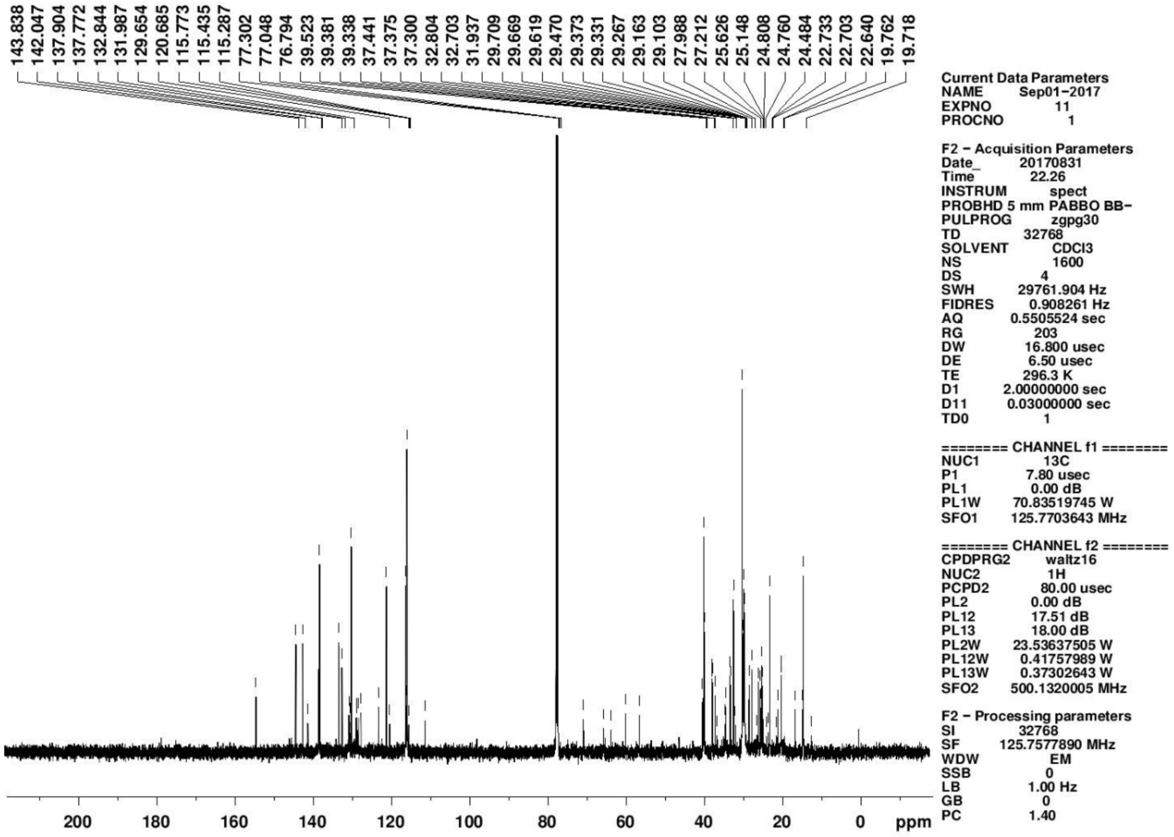

Figure S1 c. ^{13}C NMR data for APC (125 MHz). Recorded in $CDCl_3$.

Figure S2a,b.
Raw blot showing expressions of TPO and TSHR in thyroid gland of Control, APC, T_4, T_4 +APC and T_4+PTU treated animals.

Note : The gels have been run under the same experimental conditions. The membrane was cut according to the molecular weight range indicated by the marker and then incubated with the corresponding anti-bodies.

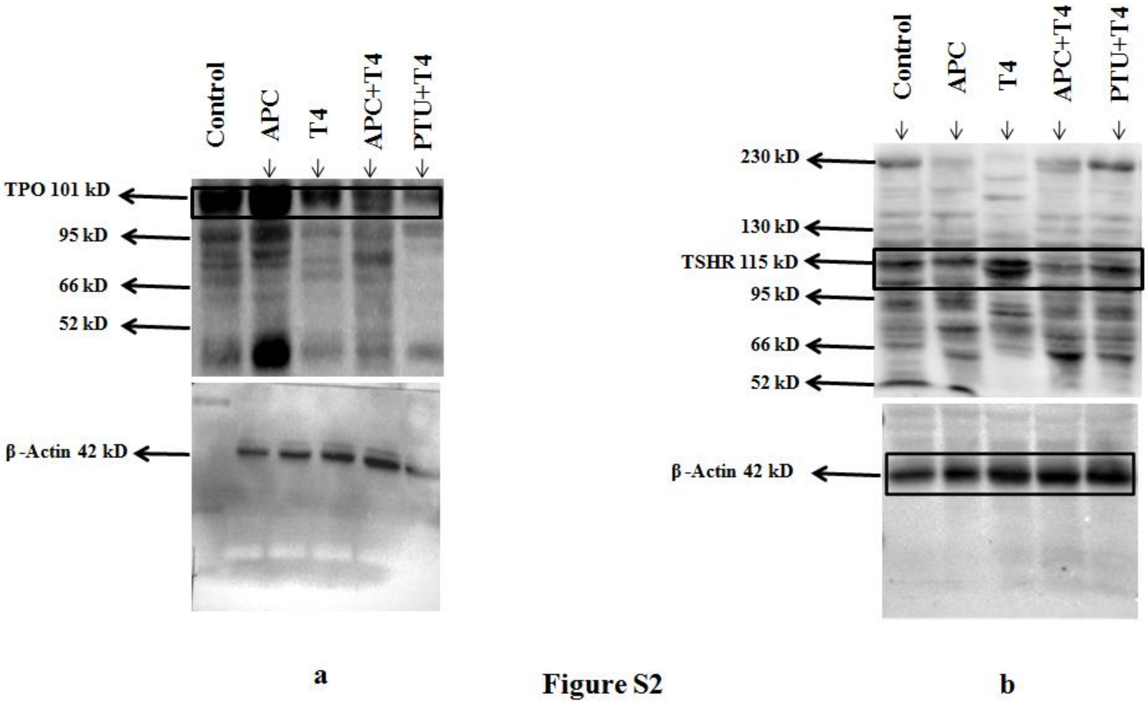

Figure S2

Raw blot images showings expression of TPO and TSHR in thyroid gland of Control, APC, T_4, T_4+APC and T_4+PTU treated animals.

Note : The gels have been run under the same experimental conditions. The membrane was cut according to the molecular weight range indicated by the marker and were incubated with the corresponding anti-bodies.
